# Transanal high pressure barotrauma causing colorectal injuries: a case series

**DOI:** 10.1186/s13256-019-2067-y

**Published:** 2019-05-07

**Authors:** Lovenish Bains, Amit Gupta, Ronal Kori, Vignesh Kumar, Daljit Kaur

**Affiliations:** 1 Department of Surgery, Maulana Azad Medical College, New Delhi, India; 2 Department of Surgery, All India Institute of Medical Sciences, Rishikesh, India; 3 Department of Trauma Surgery, All India Institute of Medical Sciences, New Delhi, India; 4 Department of Transfusion Medicine, All India Institute of Medical Sciences, Rishikesh, India

**Keywords:** Transanal, Colorectal, Barotrauma, Compressed air, Rectosigmoid

## Abstract

**Background:**

Rectal perforation by foreign bodies is known; however, high-pressure injury leading to rectal blowout has been confined to battlefields and is less often encountered in general medical practice. Apart from iatrogenic injuries during colonoscopy, barotrauma from compressed air is encountered very less frequently. Owing to the infrequent nature of these injuries, the mechanism is still not well understood. We present our experience with treating high-pressure transanal barotrauma to the rectum and colon in three similar cases.

**Case presentation:**

The mode of injury was accidental or a cruel, perverted joke played by acquaintances. The high-pressure air jet column overcomes the anal sphincter barrier, pushing enormous amounts of air through the anus into the bowel, which ruptures when the burst pressure is reached. A huge amount of free gas was noted in the peritoneal cavity on x-rays, and a big gush was noted during surgery. All these cases had rectosigmoid junction blowout with multiple colonic injuries. The patients underwent exploratory laparotomy with resection of severely injured segments and proximal ileostomy. They underwent restoration of bowel continuity after 2–3 months and were doing well in follow-up.

**Conclusions:**

Colorectal injuries by pneumatic insufflation through the anus depends on the air pressure, air flow velocity, anal resting pressure, and the distance between the source and anus. The relative fixity of the rectum and the bends of the sigmoid make the rectosigmoid junction more prone to rupture by high-pressure air jet. Education regarding such machines and their safe use must be encouraged because most of these cases are accidental and due to ignorance.

## Introduction

**Fig. 1:**
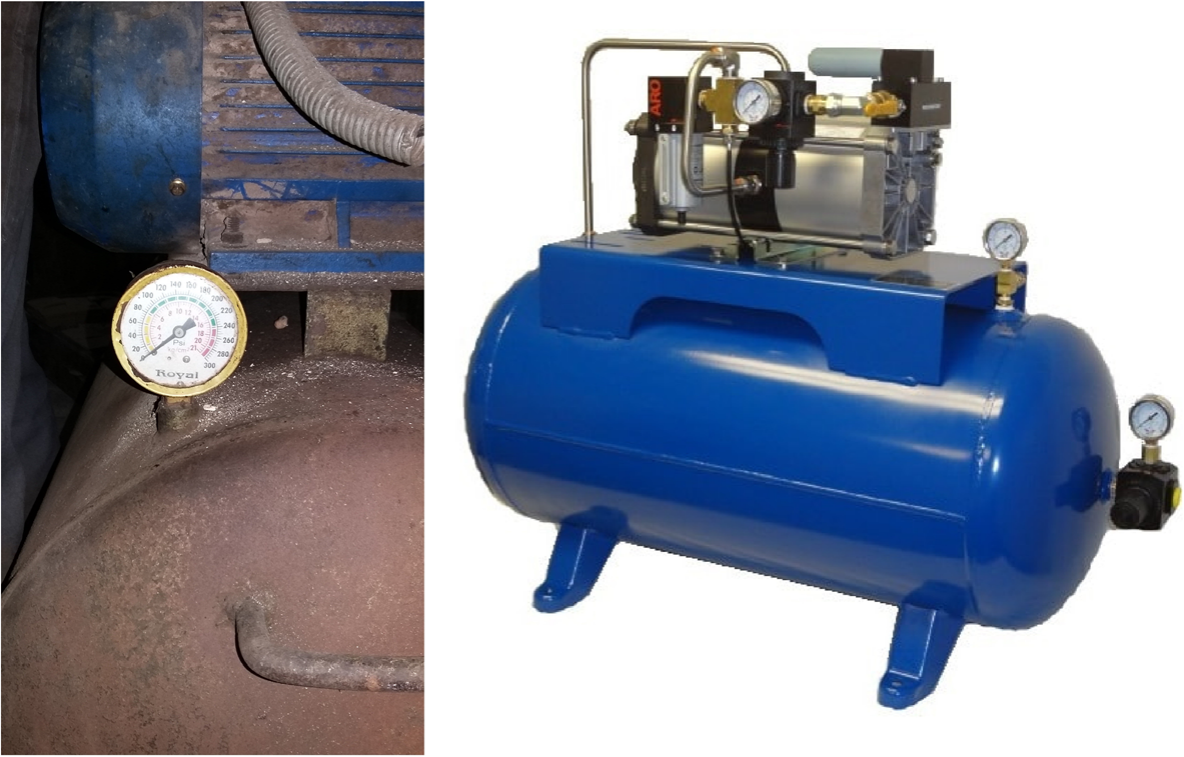
Industrial compressed air pump prototype (left one used in our patient)

With industrialization, the use of machinery has increased. One such industrial appliance is the high-pressure air compressor (Fig. 1), which is commonly used to inflate tires among many other routines. Ignorance and perversion have led to ill effects of using such machines with injuries resulting from accidental and deliberate exposure of the anus to compressed air. Barotrauma is defined as physical damage to body tissues caused by a difference in air pressure between the viscous material and its surroundings. Only a handful cases of such nature have been reported, with the first case dating back more than a century ago, reported in 1904 by Stone [1]. Injuries may vary from “cat scratch” colon [2] in mild types of iatrogenic barotrauma to colorectal perforation or blowout, the severe variety.

## Case presentation

### Case 1

**Fig. 2:**
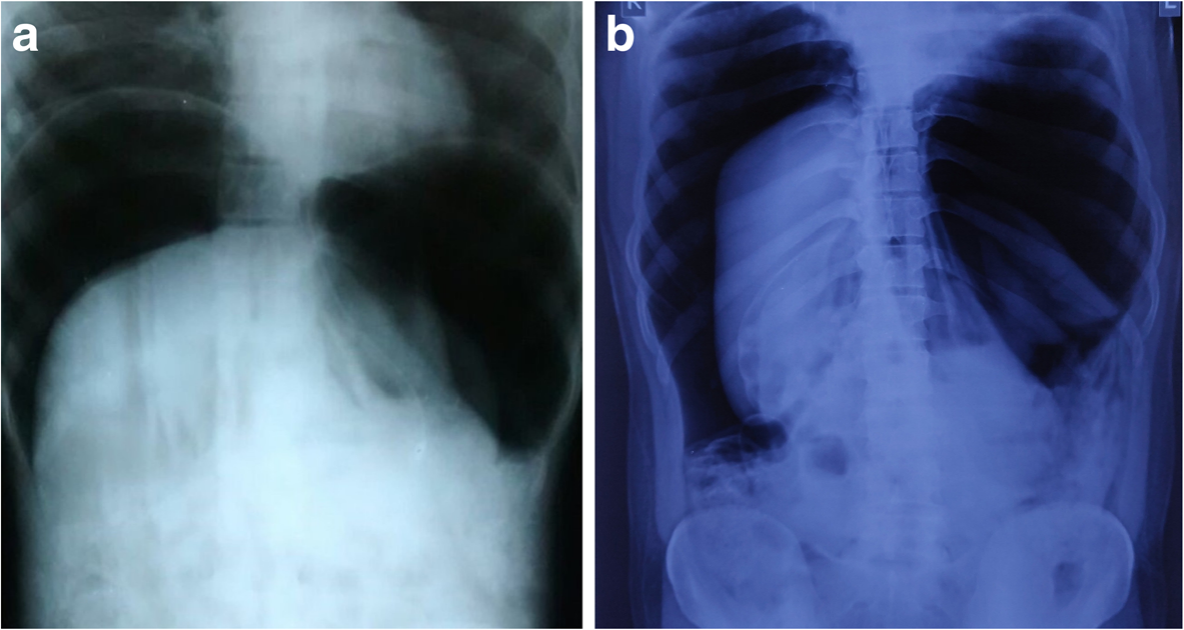
**a** and **b** Abdominal x-ray showing gross free air in peritoneal cavity

**Fig. 3:**
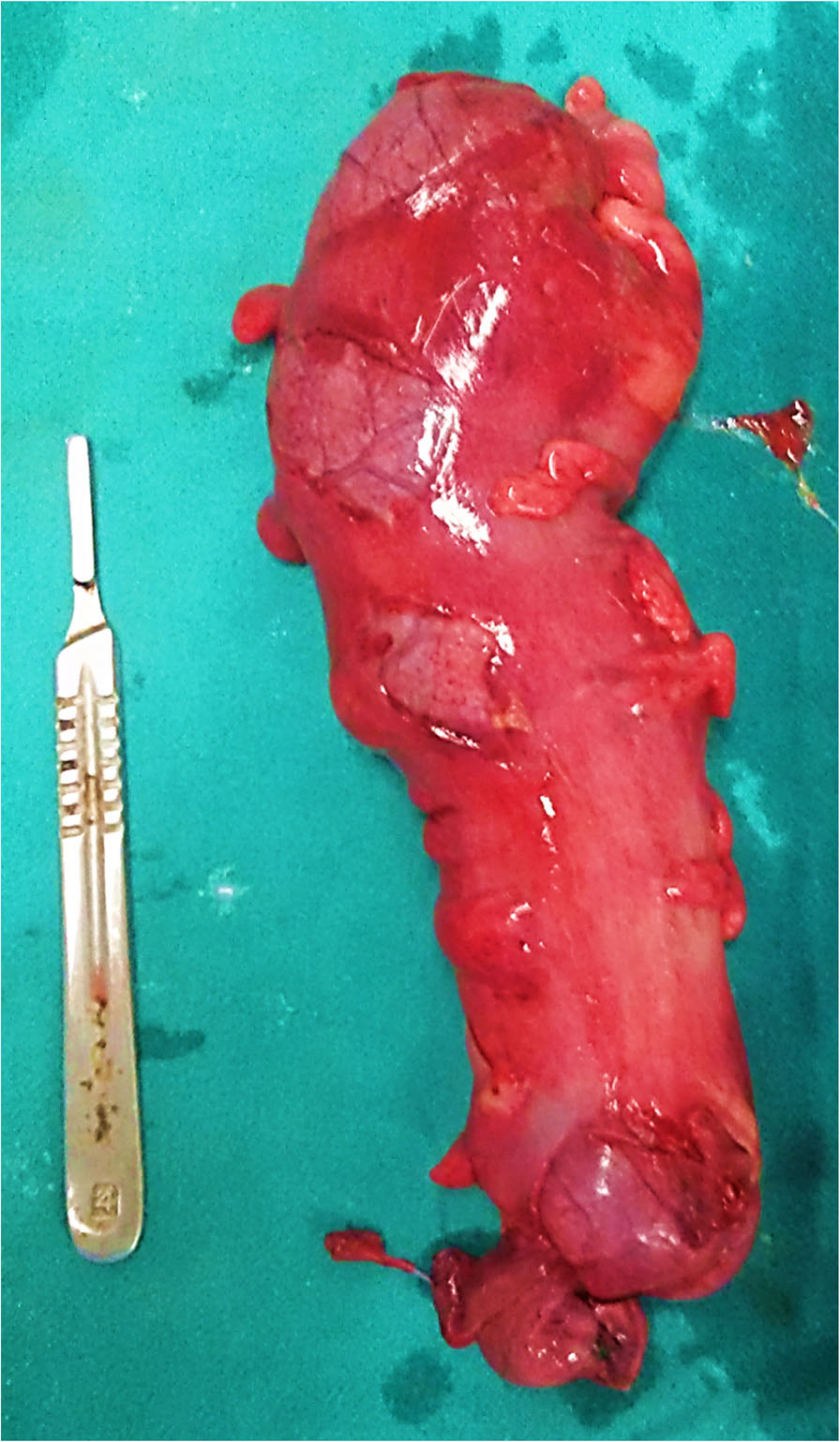
Multiple seromuscular tears in the sigmoid colon (resected specimen filled with saline)

**Fig. 4:**
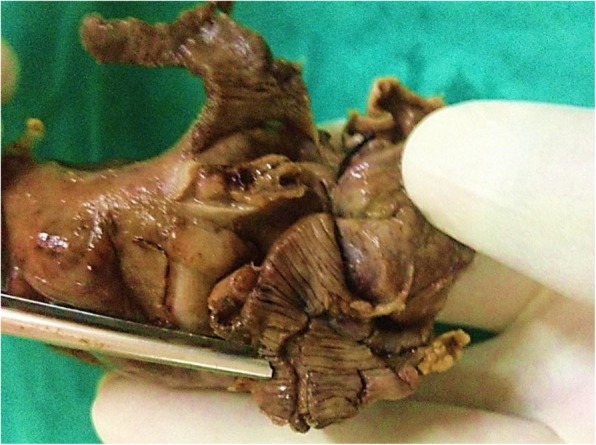
The blowout at rectosigmoid junction and ruptured muscle fibers (formalin-fixed specimen)

A 24-year-old Caucasian descent man presented to the emergency department of our institution with an alleged history of accidental injury to the anus by compressed air jet, following which he developed diffuse abdominal pain and distention. According to the patient’s history, an air nozzle was placed at a distance of approximately 25 cm from the anus for less than 1 s. At admission, his pulse rate was 96 beats/min, blood pressure 106/70 mmHg, and respiratory rate 22 breaths/min. His abdomen was distended with diffuse tenderness and tympanic on percussion with obliteration of liver dullness. His digital rectal examination was stained with blood and fecal matter. An abdominal x-ray revealed gross pneumoperitoneum (Fig. 2a). He was sent for emergency laparotomy after adequate resuscitation. On exploration, a huge gush of air was noticed. The entire peritoneal cavity was soiled with fecal matter and blood. Multiple seromuscular tears (Figs. 3 and 4) were present in the sigmoid and terminal part of the descending colon with full-thickness blowout at the rectosigmoid junction. The patient underwent resection of the sigmoid colon, closure of the distal rectal stump, and end colostomy. He had an uneventful postoperative recovery and was discharged on the fifth postoperative day. Histopathological examination revealed multiple mucosal ulcerations, submucosal hemorrhages, multiple linear muscular disruptions, and perforations as mentioned.

### Case 2

A 30-year-old Caucasian descent man presented to our institution with compressed air insult caused by his perverted friends who put the nozzle of an air pipe (tire air pump) into his anus and inflated it. At admission, his pulse rate was 130 beats/min, blood pressure 80/50 mmHg, and respiratory rate 30 breaths/min with features of diffuse peritonitis. The patient was adequately resuscitated with crystalloids and then explored. There was a huge amount of free air in the peritoneal cavity with fecal soiling of the peritoneal cavity. There were multiple colonic perforations averaging 5 mm until the ascending colon with complete blowout of the rectum. The patient underwent total colectomy with end ileostomy and closure of the rectal stump along with copious lavage.

### Case 3

**Fig. 5:**
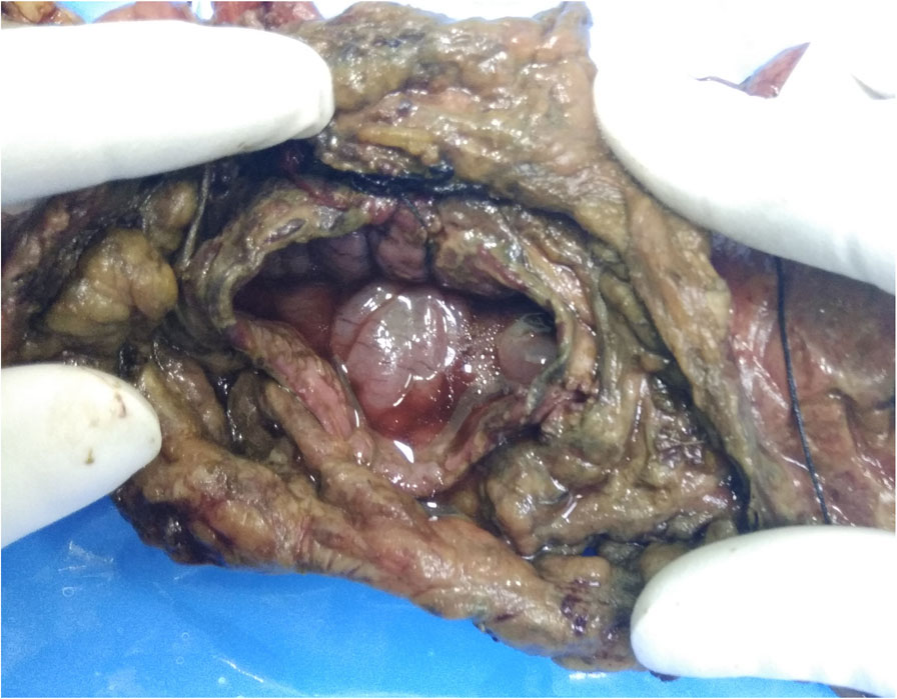
Blowout perforation in the colon

A 34-year-old Caucasian descent man, a petrol pump worker, presented 2 h after sustaining an alleged compressed air insult by robbers while thwarting the robbery. The robbers had thrust the compressed air nozzle into his anus. At admission, the patient was anxious with a pulse rate of 114 beats/min, blood pressure 124/76 mmHg, and respiratory rate 26 breaths/min. He had a distended abdomen with diffuse tenderness suggestive of peritonitis. An abdominal x-ray revealed massive pneumoperitoneum (Fig. 2b). This patient had a partial anal tear, and, on exploration, a rectal blowout at the rectosigmoid junction and multiple descending colonic perforations were noted (Fig. 5). The patient underwent resection of the descending and sigmoid colon with end transverse colostomy and rectal stump closure. The anastomosis to restore bowel continuity was done 2–3 months later in these cases after radiological assessment and anal tone assessment by anal manometry.

## Discussion

Barotrauma following colonoscopy is not uncommon, with an incidence of 0.1–0.5% [3–5]. However, colonic injuries caused by high-pressure air compressor are extremely rare and reported especially among industrial workers. During gradual insufflation of the colon or in large bowel obstruction distal to the cecum, the cecum is the segment most prone to distention injury, which is explained by the law of Laplace. The cecum has the largest diameter and hence requires the least amount of pressure to distend.

An important corollary to Laplace’s law is that the degree of angulations (sharpness of cylinder curvature) is more important than the intramural pressure in determining wall tension [6]. The anatomical configuration of the buttocks and perineum is like a funnel; it allows easy delivery of compressed air into the anal orifice. Thus, an air jet enters the anus more readily than the examining finger or proctoscope as it passes through clothes and the anus even when not accurately directed [7]. The anatomy of the distal colon with the firm lateral support of the rectum makes the rectosigmoid junction the first part of the colon to be struck by a column of pressure from an external source and the bending of the sigmoid contributes along with the rectosigmoid junction to perforation in pressure-related colon barotrauma [5].

Experimental studies have shown that the human colon bursts with only about 120–200 mmHg of pressure and that seromuscular rupture occurs at lower levels [8]. Through colonoscopic studies, it has been estimated that the intraluminal pressure required to result in colon perforation is greater than 0.109 kg/cm^2^ (1.547 psi/80 mmHg) [9]. However, colonic injuries following high-pressure barotrauma result in injuries elsewhere in the colon, predominantly in the rectosigmoid junction. The sudden nature of the insult does not allow air to uniformly pass through the entire colon, and the outcome is a perforation of the rectosigmoid junction. Industrial air compressors can be classified according to the pressure delivered as low-pressure air compressors (discharge pressure of 150 psi or less), medium-pressure compressors (151 psi to 1000 psi), and high-pressure air compressors (above 1000 psi). Mostly, low-pressure or medium-pressure compressors are used.

The resting pressure of the anal sphincter is 40–80 mmHg. The air flow of compressed air jet in use is estimated to be 141 L/min (5 cfm), which is 100-fold greater than the safe level airflow of 1.46 L/min (at 80 mmHg intraluminal pressure) during colonoscopic examination; such an air thrust exceeds the resting anal pressure, overcomes the anal sphincter pressure, and results in sudden inflation of the colon [3, 4]. Andrews postulated that air at 3.5–8.8 kg/cm^2^ (50–125 psi/2585–6464 mmHg) forms a column that acts like a solid body, forcing open the anal sphincter [10]. It takes only 1 or 2 s to deliver enough pressurized air to cause major damage. It has been estimated that a pressure of 0.27 kg/cm^2^ (3.99 psi/201.69 mmHg) is required to rupture the serosa and the muscles of the intestinal wall, and a pressure of 0.29 kg/cm^2^ (4.07 psi/210.48 mmHg) will cause a through-and-through rupture [11]. Perforations distal to the rectosigmoid junction have seldom been reported. This may be because the rectum and anus are well supported by pelvic structures and the bilateral fixity of the rectosigmoid junction. Thus, injury as a result of high-pressure barotrauma depends on the air pressure, air flow velocity, anal resting pressure, and the distance between the source and anus [12–15]. Management ranges from expectant management to a formal laparotomy. Individuals with no clinical or radiological signs of peritonitis can be managed expectantly, whereas signs of peritonitis, if present, indicate the need for operative intervention. Surgical interventions for pneumatic colon injury include rectal tube decompression, intraoperative decompression of bowel in the presence of distended bowel, resection of severely injured segment of colon, and repair of perforation with proximal diverting colostomy or enterostomy, when the integrity of the bowel is in doubt [14–16]. Careful observation following surgery is often necessary because full-thickness perforation of the colon may have a delayed presentation.

Sudden entry of pressurized air may rarely lead to a tension pneumoperitoneum. The difference between simple pneumoperitoneum and tension pneumoperitoneum is the presence of enormous tension in the peritoneal space, which can have fatal hemodynamic and respiratory compromise [7, 17]. Decreased venous return to the heart due to compression of the inferior vena cava and splanchnic circulation results in hypotension and may lead to shock. Elevation of the diaphragm due to pressure from below decreases the lung volume affecting ventilation. Unless promptly treated, death may occur due to air embolism, fat embolism, respiratory insufficiency due to high intra-abdominal pressure and chest compression, and acute heart failure due to deficient preload and peritoneal shock, hyperacute abdominal compartment syndrome. It may also cause compression of the aorta, of mesenteric vessels leading to bowel ischemia, or rarely venous congestion and ischemia of lower extremities [18]. Timely intervention is required in such cases. Management is converting a tension pneumoperitoneum into an open pneumoperitoneum akin to tension pneumothorax. Urgent percutaneous decompression of the tension pneumothorax by means of a cannula, a trocar, or a Veress needle is a simple, quick, and useful method.

## Conclusions

Colonic barotrauma due to compressed air may happen as a result of perversion or accidental injury in industrial zones. Education regarding such machines and their safe use must be encouraged because most of these cases are accidental and due to ignorance. The amount of injury may vary from mucosal ulceration to full-thickness blowout, depending on air pressure, air flow velocity, anal resting pressure, and the distance between the source and anus. Management ranges from repair or resection with proximal enterostomy, because multiple other injuries may perforate later and have a delayed presentation.
